# Ultrasound confirmation of guidewire position may eliminate accidental arterial dilatation during central venous cannulation

**DOI:** 10.1186/1757-7241-18-39

**Published:** 2010-07-13

**Authors:** Lawrence M Gillman, Michael Blaivas, Jason Lord, Azzam Al-Kadi, Andrew W Kirkpatrick

**Affiliations:** 1 Departments of Surgery, University of Calgary, (1403 - 29 Street NW), Calgary, (T2N 2T9), Canada; 2 Department of Critical Care Medicine, University of Calgary, (1403 - 29 Street NW), Calgary, (T2N 2T9), Canada; 3 Regional Trauma Services, University of Calgary, (1403 - 29 Street NW), Calgary, (T2N 2T9), Canada; 4 Division of Emergency Medicine, University of Calgary, (1403 - 29 Street NW), Calgary, (T2N 2T9), Canada; 5 Department of Emergency Medicine, Northside Hospital Forsyth, (1200 Northside Forsyth Drive), Cumming, (30041), USA

## Abstract

**Background:**

Ultrasound guidance during central line insertion has significantly reduced complications associated with this procedure and has led to it being incorporated as standard of care in many institutions. However, inadvertent arterial penetration and dilation remains a problem despite ultrasound guidance and can result in significant morbidity and even mortality. Dynamic ultrasound confirmation of guidewire position within the vein prior to dilation may help to prevent and even eliminate this feared complication.

**Methods:**

A prospectively collected database of central line insertions for one author utilizing this novel technique was retrospectively reviewed for all incidents of arterial dilation over a period from September 2008 to January 2010.

**Results:**

During the study period 53 central lines were inserted with no incidents of arterial dilation.

**Conclusions:**

Ultrasound confirmation of guidewire position has the potential to reduce or eliminate the morbidity and mortality of arterial dilation during central line placement.

## Background

Traditionally central lines were placed blindly using only anatomical landmarks along with proper patient positioning to approximate the location of the appropriate vessel. Adjunct techniques such as utilization of small gauge finder needles may have lowered the incidence of arterial penetration and cannulation, but accidental arterial cannulation remained a significant problem. More recently ultrasound has made a move from the radiology department to the bedside, and has been integrated into many bedside procedures. Currently, jugular and femoral central lines in many institutions are now being inserted under dynamic ultrasound guidance. This is a result of multiple studies suggesting decreased complications and improved success rate when ultrasound was utilized [1-4]. Nevertheless, this dynamic technique has not completely eliminated all insertion complications. A recent paper, reported on a series of arterial dilations despite direct ultrasound guidance during the procedure [5]. These complications were associated with significant morbidity and even mortality. This may be potentially preventable if the guidewire can be visualized clearly within the vein with ultrasound prior to dilation. To our knowledge this is the first reported study utilizing this method to prevent unplanned arterial dilations during central line placement.

## Methods

### Subjects

All adult patients admitted to two multi-system intensive care units in the Calgary Health Region requiring either internal jugular or femoral venous access that was performed under ultrasound guidance by one author.

### Study design

A database of all central line insertions in the Calgary Health Region, Department of Critical Care over a period from September 2008 to January 2010 was retrospectively reviewed as part of a quality assurance initiative. All internal jugular and femoral lines inserted by one author (LG) were captured, and reviewed.

### Protocol

**Figure 1:**
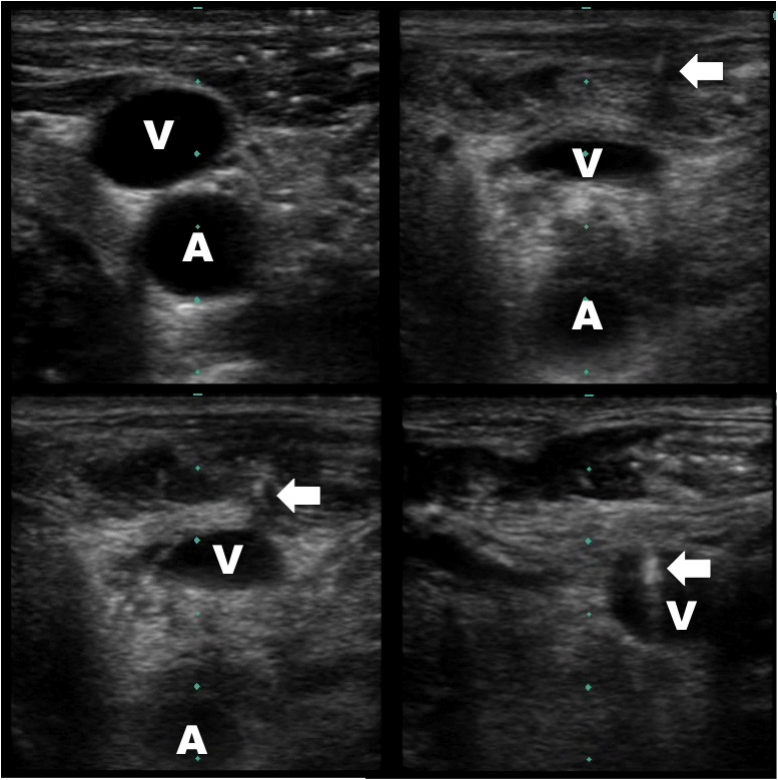
**Guidewire within jugular vein - short axis**. Ultrasound guided placement of a left internal jugular central line. The guidewire (arrow) can be seen within the internal jugular vein (V) lateral and superficial to the artery (A) in the short axis.

**Figure 2:**
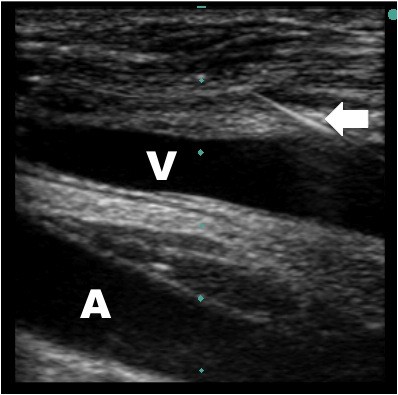
**Guidewire within jugular vein - long axis**. Ultrasound guided placement of a left internal jugular central line. The guidewire (arrow) can be seen within the internal jugular vein (V) lateral and superficial to the artery (A) in the long axis.

**Figure 3:**
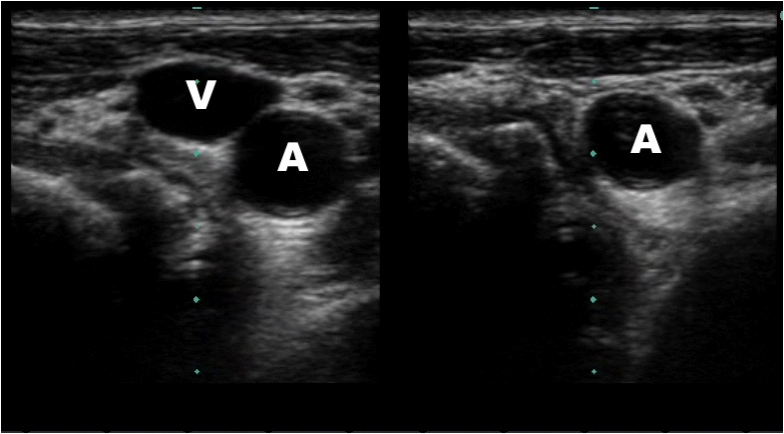
**Identification of jugular vein by obliteration with pressure**. Ultrasound guided placement of a left internal jugular central line. The artery and vein are differentiated by the complete obliteration of the vein (V) with compression compared with the artery (A).

**Figure 4:**
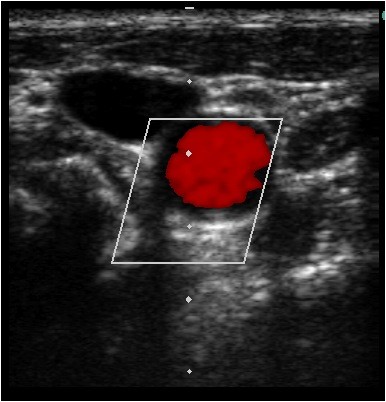
**Identification of carotid artery by color flow Doppler**. Ultrasound guided placement of a left internal jugular central line. The artery and vein are differentiated by color flow Doppler confirming pulsatile flow within the artery and not the vein.

All veins were cannulated using a standard Seldinger technique under direct ultrasound guidance in the short axis using either a Sonosite MicroMaxx or M Turbo (Sonosite Corporation, Bothell, WA). Once the guidewire was successfully placed intravascular, its position within the vein was confirmed by direct visualization with ultrasound along its entire visible course (Figure 1, 2, Additional File 1, 2). The artery and vein were differentiated by complete obliteration of the vein with compression compared with the artery (Figure 3, Additional File 3) and by pulsation of the artery (additional FIle 4). If differentiation was still in question, color flow Doppler was used to confirm pulsatile flow within the artery and not the vein (Figure 4, Additional File 5). The tract was subsequently dilated and the central line placed. Venous placement of all lines was then confirmed by pressure transduction, blood gas analysis or both. All complications were logged in the prospective database. All incidents of arterial dilatation were recorded.

## Results

During the 16 month study period 53 central lines were inserted by LG: 21 central venous introducers, 9 dialysis catheters and 23 multi-lumen venous catheters. There were no incidents of accidental arterial puncture or arterial dilation during the study period.

## Discussion

Ultrasound guidance is quickly becoming accepted as a standard of care for central line insertion in many institutions and is supported by the Agency for Healthcare Research and Quality [6]. Not only does it result in a significant reduction in complications as well as greater first-pass success [1,2,4,7], it can also be used to confirm proper guidewire position and post line insertion to screen for complications such as pneumothorax [8]. In an extensive meta-analysis, ultrasound guidance significantly reduced internal jugular and subclavian placement failure (relative risk [RR] 0.32; 95% confidence interval [CI] 0.18-0.55), decreased complications during catheter placement (RR 0.22; 95% CI 0.18-0.55) and lowered the need for multiple catheter placement attempts (RR 0.6; 95% CI 0.45-0.79) when compared with the standard landmark placement technique [1].

Ultrasound guided line access is not limited to central line access but it can be utilized for difficult peripheral line access [9]. Brannam et al. reported a high success rate and low complication rate for US-guided peripheral vascular access performed by Emergency Room nurses with minimal US training [10].

While arterial puncture during insertion is often well tolerated, arterial dilation and cannulation may carry significant morbidity and potential mortality [5,11-13]. The Australian Incident Monitoring Study has reported that 50% of central line incident reports are due to inadvertent arterial injuries which have led to increase patient morbidity and/or prolonged hospital stay [14]. Even under direct ultrasound guidance using either the short or long axis if the needle passes outside of the ultrasound beam, the tip may actually be deeper than suspected and may pass through the posterior wall of the vein [15] into deeper unseen structures such as the carotid artery. Thus, the unsuspecting clinician may proceed with dilation being unaware that he/she is actually penetrating the incorrect vessel.

Clinical measures used to confirm venous versus arterial needle location include the presence of pulsatile flow and bright red blood. However, these may be unreliable or difficult to interpret in hypotensive and hypoxic critically ill patients. Other methods, such as needle transduction and blood gas analysis are time-consuming, costly and may significantly delay central line placement.

Our method utilizes dynamic ultrasound visualization of the guidewire within the vein along its entire course as a method to confirm intravenous guidewire placement prior to dilation. In this way the guide wire can be followed from the point of insertion until well past the needle puncture site in the vein in both the short (Figure 1, Additional File 1) and long axis (Figure 2, Additional File 2). Unless the patient is in cardiac arrest or profoundly hypotensive, the artery and vein should be easily differentiated by complete obliteration of the vein with compression compared with the artery (Figure 3, Additional File 3) and by pulsation of the artery (Additional File 4). If differentiation is still unclear, color flow Doppler can be used to confirm pulsatile flow within the artery and not the vein (Figure 4, Additional File 5). This method is fast, easily taught and if ultrasound guidance is already being used for central line placement does not require any additional equipment. To our knowledge this is the first reported case series utilizing this method to prevent arterial dilation. In this study, there were no arterial dilations in a series of 53 central line insertions.

There are, however, multiple limitations to this study. This area is poorly studied in the literature to date and the true incidence of arterial dilation during central line insertion is unknown. It is possible that this study is underpowered and this may explain the absence of complications during the study period. In addition, this represents the experience of a single physician who is extensively trained and practiced in central line insertion. This technique requires further study in a variety of settings, by operators with a range of skill and experience before comments on its generalizability can be made.

## Conclusions

Ultrasound confirmation of guidewire position is a simple, easy to use technique that requires no new training or equipment. This study suggests that it has the potential to reduce or eliminate the morbidity and mortality of arterial dilation during central line placement and clearly warrants further consideration and study. Line insertion under ultrasound guidance may produce a false sense of security and we submit that this procedure may serve as a “reality check” prior to dilation and cannulation.

## Competing interests

The authors declare that they have no competing interests.

## Authors' contributions

LG, AK, JL and MB conceived of the study, and participated in its design and coordination. LG and AA were involved in data collection and helped to draft the manuscript. LG and AK were involved in image acquisition and editing. All authors read and approved the final manuscript.

## Supplementary Material

Additional file 1 **Guidewire within jugular vein - short axis**. Ultrasound guided placement of a left internal jugular central line. The hyperechoic guidewire can be seen within the internal jugular vein along its entire visible course in the short axis. Click here for file

Additional file 2 **Guidewire within jugular vein - long axis**. Ultrasound guided placement of a left internal jugular central line. The hyperechoic guidewire can be seen within the internal jugular vein along its entire visible course in the long axis. Click here for file

Additional file 3 **Identification of jugular vein by obliteration with pressure**. Ultrasound guided placement of a left internal jugular central line. The artery and vein are differentiated by the complete obliteration of the vein with compression compared with the artery. Click here for file

Additional file 4 **Identification of carotid artery by pulsation**. Ultrasound guided placement of a left internal jugular central line. The artery and vein are differentiated by the pulsation within the carotid artery. Click here for file

Additional file 5 **Identification of carotid artery by color flow Doppler**. Ultrasound guided placement of a left internal jugular central line. The artery and vein are differentiated by color flow Doppler confirming pulsatile flow within the artery and not the vein. Click here for file
